# Harmonic Components Analysis of Emitted Ultraviolet Signals of Aged Transmission Line Insulators under Different Surface Discharge Intensities

**DOI:** 10.3390/s22030722

**Published:** 2022-01-18

**Authors:** Saiful Mohammad Iezham Suhaimi, Nor Asiah Muhamad, Nouruddeen Bashir, Mohamad Kamarol Mohd Jamil, Mohd Nazri Abdul Rahman

**Affiliations:** 1 School of Electrical and Electronic Engineering, Engineering Campus, Universiti Sains Malaysia, Nibong Tebal 14300, Malaysia; saifuliezham@student.usm.my (S.M.I.S.); eekamarol@usm.my (M.K.M.J.); 2 Faculty of Engineering, Universiti Teknologi Brunei, Gadong BE1410, Brunei; 3 Department of Electrical Engineering, Faculty of Engineering, Kano University of Science and Technology, Wudil P.M.B. 3244, Kano State, Nigeria; 4 Faculty of Education, University of Malaya, Kuala Lumpur 50603, Malaysia; mohdnazri_ar@um.edu.my

**Keywords:** transmission line insulators, discharge intensity level, ultraviolet pulse (UVP), contamination level

## Abstract

Flashover on transmission line insulators is one of the major causes of line outages due to contamination from the environment or ageing. Power utility companies practicing predictive maintenance are currently exploring novel non-contact methods to monitor insulator surface discharge activities to prevent flashover. This paper presents an investigation on the UV pulse signals detected using UV pulse sensor due to the discharges on the insulator surfaces under varying contamination levels and insulator ages. Unaged and naturally aged insulators (0 to >20 years) were artificially contaminated (none, light to heavy contamination). The electrical stresses on the insulator surfaces were varied to generate varying discharge intensity levels on the surfaces of the insulator. The DC and harmonic components of UV pulse signals detected during surface discharges were recorded and analysed. Results show a positive correlation between the discharge intensity level of contaminated and aged transmission insulators with the DC and harmonic components of the UV pulse signals. Furthermore, the study revealed that under dry insulator surface conditions, insulator ageing has a more profound effect during discharges than contamination level. The findings from this study suggest that the use of UV pulse sensors to monitor UV pulse signals emitted during insulator surface discharges can be another novel non-contact method of monitoring transmission line insulator surface conditions.

## 1. Introduction

Currently, discharge detection methods for condition monitoring/maintenance of transmission line insulators are widely used and have shown to be effective and reliable. Discharges on insulators occur if their surfaces are contaminated or become less hydrophobic due to various environmental factors at the vicinity of a line [1]. These environmental factors lower the insulator surface resistance leading to a higher flow of leakage current. If this phenomenon exacerbates, a flashover could occur that can lead to an outage. Discharges on insulator surfaces are precursors to contamination flashover.

Several methods have been proposed for the monitoring of discharge intensity levels on the insulator surfaces by researchers [2,3,4] to ensure convenient, reliable, and accurate monitoring and prediction of insulator surface conditions. Currently, there has been more focus on non-contact methods of monitoring insulator surfaces for convenience and reliability. Such methods include acoustic, thermal [2,5,6], UV intensity, infrared [7,8], etc.

Among such methods, the UV method has been reported to be very promising due to its reliability in field measurements. Corona discharges emit UV radiations at wavebands between 280 nm–400 nm, and a few others range from 160 nm to 180 nm [9]. Most studies on the UV method involve UV Pulse (UVP) method and UV Image (UVI) method with a focus on the latter [8,9,10,11,12,13]. The UVI method employs a UV camera to capture and produce a UV image of the discharge to estimate its intensity, while the UVP method uses a UVP sensor to detect the intensity level of UV signals produced by the discharge on the insulator surfaces.

Studies have shown a good correlation between the DC components of UV signals and the UV intensity. In a study conducted by [14], correlations were reported between the UV intensity and DC signals. GaN UV sensor with three kinds of different film (conductive, semi-conductive, and insulating) was used in the study. As the intensity level of the signals increases, the DC signals also increase. DC components are the resultant signals at zero frequency. Theoretically, a signal is said to be purely resistive at zero frequency.

The objective of this present work is to investigate the correlation between the discharge intensity levels of contaminated and aged transmission insulators with the DC and harmonic components of UV pulse signals detected using UV pulse sensor due to the discharge activities on the insulator surfaces under varying contamination levels and insulator ages.

## 2. DC Components and Harmonics of Periodic Signals

When a periodic signal is distorted, it possesses harmonic contents. The frequency of the periodic signal is known as the fundamental frequency, and the harmonics are the signals whose frequency is an integer multiple of the fundamental frequency. A general function of a periodic signal *x*(*t*) is given by Equation (1):

(1) $$x\left(t\right)=A\sin\left(2\pi ft+\varphi \right)$$

where *A* is the amplitude, *f* frequency, and *φ* phase.

**Figure 1:**
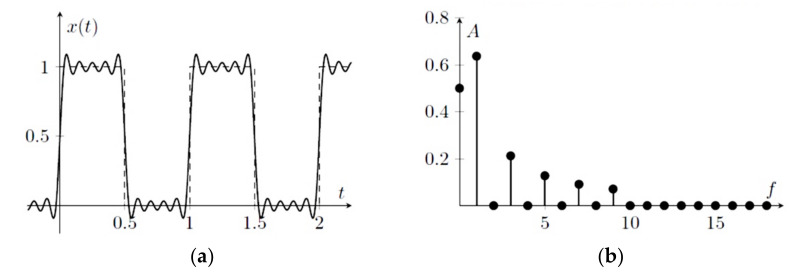
Distorted square wave representation: (**a**) time-domain; (**b**) frequency-domain [15].

To analyse the harmonics in a distorted signal, the magnitudes and phases of the fundamental and higher-order harmonics are calculated using the Fourier series expressed by Equation (2) represented in frequency-domain.

(2) $$x\left(t\right)=A_{o}+\overset{\infty }{\underset{n=1}{\sum }}A_{n}\sin\left(2\pi ft+\varphi_{n}\right)$$

where $A_{o}$ is called the DC component or DC offset, $A_{n}$ are higher-order harmonics having phases $\varphi_{n}$. Equation (2) expresses the periodic signal $x\left(t\right)$ as a sum of sinusoidal signals having different amplitudes ($A_{o},\;A_{1},\;A_{2}\ldots \ldots \;A_{n}$) and phases ($A_{o},\;\varphi_{1},\;\varphi_{2}\ldots \ldots \;\varphi_{n}$). The DC component is the zero-frequency component, plotted at 0 Hz in the frequency-domain representation of a periodic signal. The harmonics have *f*, 2*f*, 3*f*, 4*f*, and so on, with *f* referred to as the fundamental frequency. Figure 1 illustrates the time-domain and frequency-domain representations of a distorted square wave. The signal has a zero-frequency component (DC component) of the magnitude of 0.5.

## 3. Methodology

### 3.1. Experiment Setup

**Figure 2:**
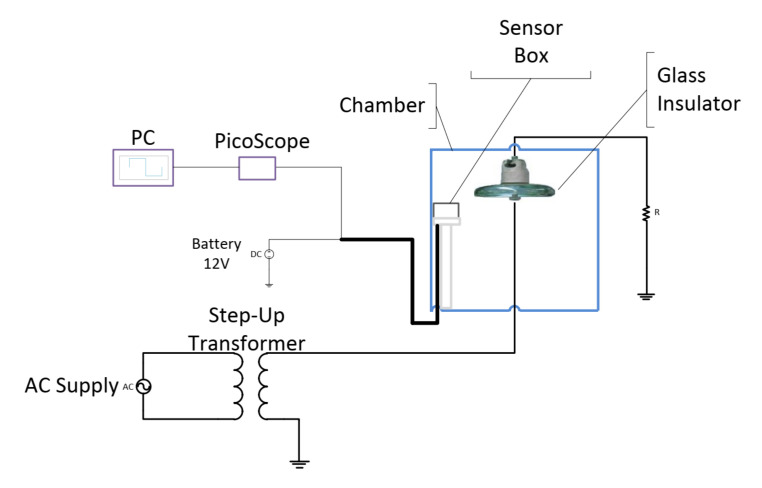
Schematic diagram for the overall experiment setup.

Figure 2 depicts the experimental setup to conduct the experiments to achieve the objectives of this study. The experimental setup consists of a chamber made of acrylic glass, a UVP sensor, a high voltage source, and a Personal Computer (PC) for data acquisition, storage, and analyses. The high voltage source consists of a 100 kV step-up transformer with a voltage regulator for controlling the applied voltage to the insulator sample. The UVP sensor was placed directly facing the insulator sample under test to detect the UV signals emitted by the discharges on the insulator surface. The output of the UVP sensor was connected to a PicoScope (digital oscilloscope) so that the UV pulse signals could be captured by the PC, which is connected to the output of the PicoScope. The UVP sensor is powered by a 12 V DC supply.

In order to investigate the surface discharges of the insulators at various intensities, four intensity levels were studied. These intensities were produced by increasing the electric stress on the insulator. To vary the electric stress on the insulator surface, the applied voltage was increased until the desired discharge intensity level was obtained. Once the desired level was reached, measurements were taken. Table 1 shows the discharge intensity levels produced in this study and their characteristics.

**Figure 3:**
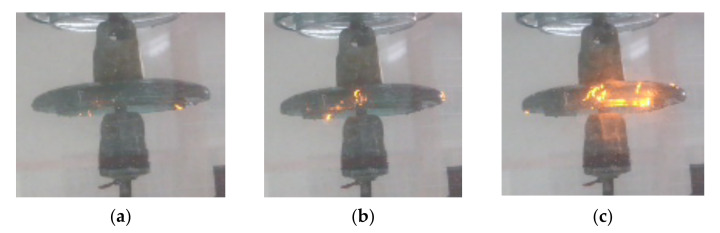
Pictorial view of generated discharges: (**a**) at pin (**b**) at cap and pin (**c**) severe.

Pictures of the generated different discharge intensities is shown in Figure 3.

### 3.2. Ultra Violet Pulse (UVP) Sensor

**Figure 4:**
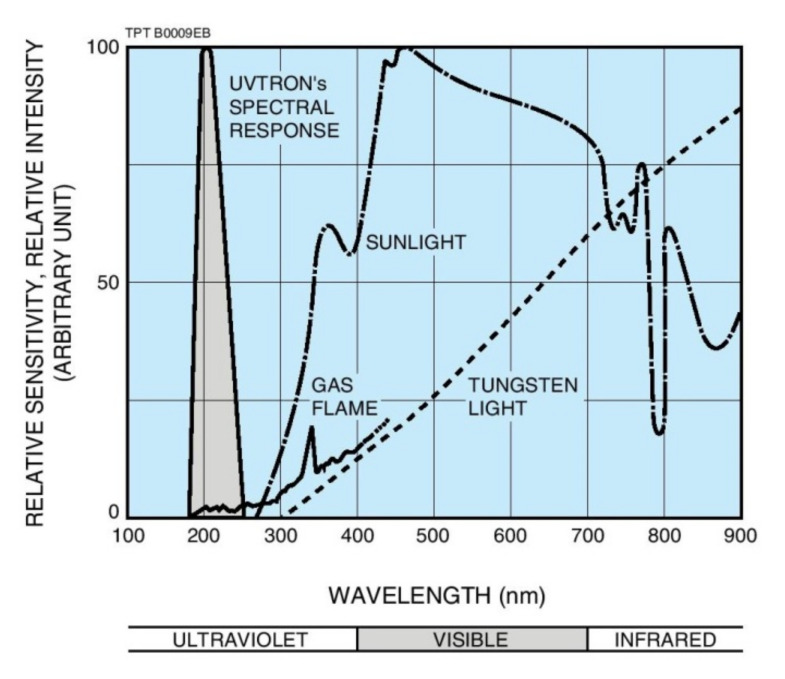
Comparison between discharge ultraviolet spectrum and visible light spectrum [16].

As mentioned in the previous section, discharge produced on the insulator surfaces emitted UV radiations [11]. The UVP sensor was used to detect and measure the UV signals. The sensor was placed approximately 1 m away from the insulator sample under test. The 1 m distance was adequate to detect the UV signals and prevent damage due to the presence of high voltage/discharges. The UVP sensor has a buzzer. The buzzing sound from the buzzer increases with an increase in the discharge intensity level. The buzzer acts as an indicator when UV signals are detected due to discharge activities on the sample insulator surface. In this study, the UV sensor used to detect the UV signals during discharges was the UVTRON R2868 manufactured by Hamamatsu Company. The sensor specification is shown in Table 2. The wavelength of UV signals the sensor is able to detect is between 160 nm to 280 nm. The sensor comes along with its driving circuit, UVTRON Driving Circuit C3704. The driving circuit activates the sensor in the event of a discharge. Figure 4 shows the response range of the UVTRON sensor.

### 3.3. Insulator Samples

#### 3.3.1. Aged Insulator

Service aged insulators were used in this study. The aged insulator samples were obtained from the National Power Utility Company of Malaysia, Tenaga Nasional Berhad (TNB). The insulators were in service from the range of <10 years to >20 years (close to 30 years) on 132 kV transmission lines having varying degrees of degradation. Descriptions of the insulator samples are presented in Table 3. These insulators have been grouped into three based on their age/length of service in the field, as shown in Table 3. It can be seen that the insulators that have been in service for less than 10 years look relatively new, not exhibiting any form of degradation. However, the insulators aged between 10 to 20 years old have exhibited mild corrosion on the insulator caps, while the insulators that were in service for more than 20 years have exhibited severe corrosion on the cap and discoloration of glass.

#### 3.3.2. Contamination Level

In this study, the insulator samples’ DC and harmonic components of the UV signals were studied under varying surface resistances by contaminating the insulator samples’ surface with different levels of contamination. The insulator samples were sprayed with saltwater having different salt amounts. The contamination levels were determined by using Equivalent Salt Deposit Density (ESDD) method [17]. The insulators were left to dry in the sun for 6 h after contamination before the commencement of the experiment. Table 4 shows the artificial contamination level considered in this study. Four contamination levels were considered, as shown in Table 4. The contamination level classified as ‘none’ in the table refers to a clean insulator surface (uncontaminated).

### 3.4. Determination of DC Component from UVP Signal

**Figure 5:**
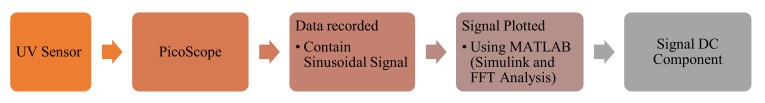
Flow to determine the DC Component of the UVP Signals.

The UVP sensor produces signals commensurate with the level of discharge intensity on the insulator’s surface. The data stored on the PC were in excel files format for ease of plot in MATLAB software. The PicoScope, which acted as a digital oscilloscope, produced adequate plotting points of the signal from the UVP sensor output. Fast Fourier Transform (FFT) analysis in MATLAB software was used to analyse the DC component of the signals. Figure 5 shows the process flow for determining the DC components of the UVP signals analysed in this work.

## 4. Results and Discussion

**Figure 6:**
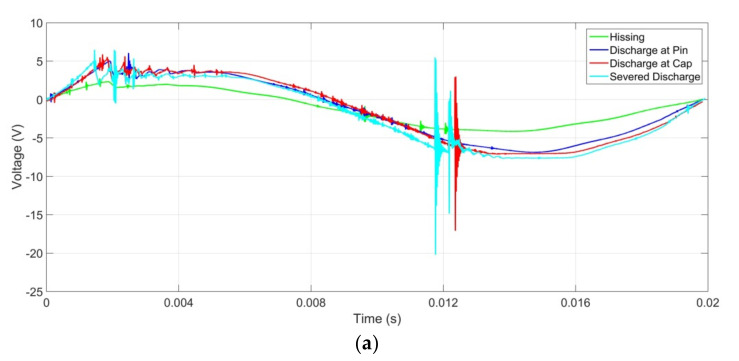

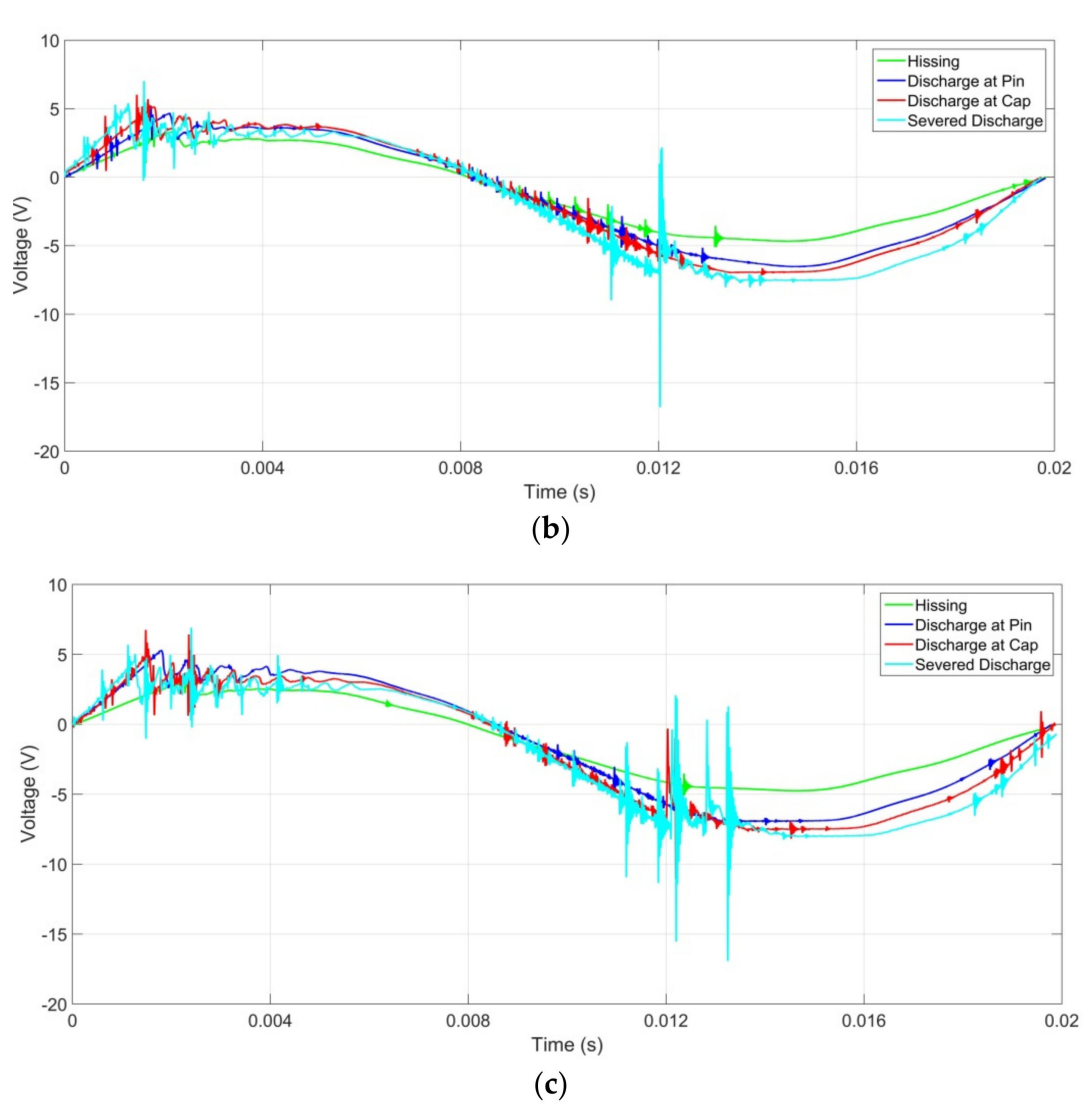
UV signal for insulator samples at light contamination level with insulator ages of (**a**) <10 years, (**b**) 10 to 20 years, and (**c**) >20 years.

**Figure 7:**
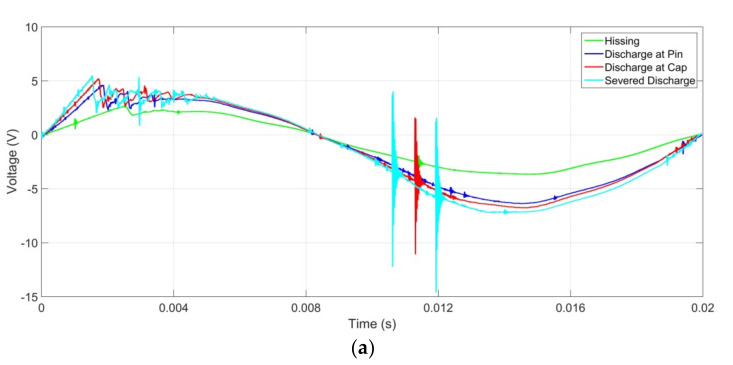

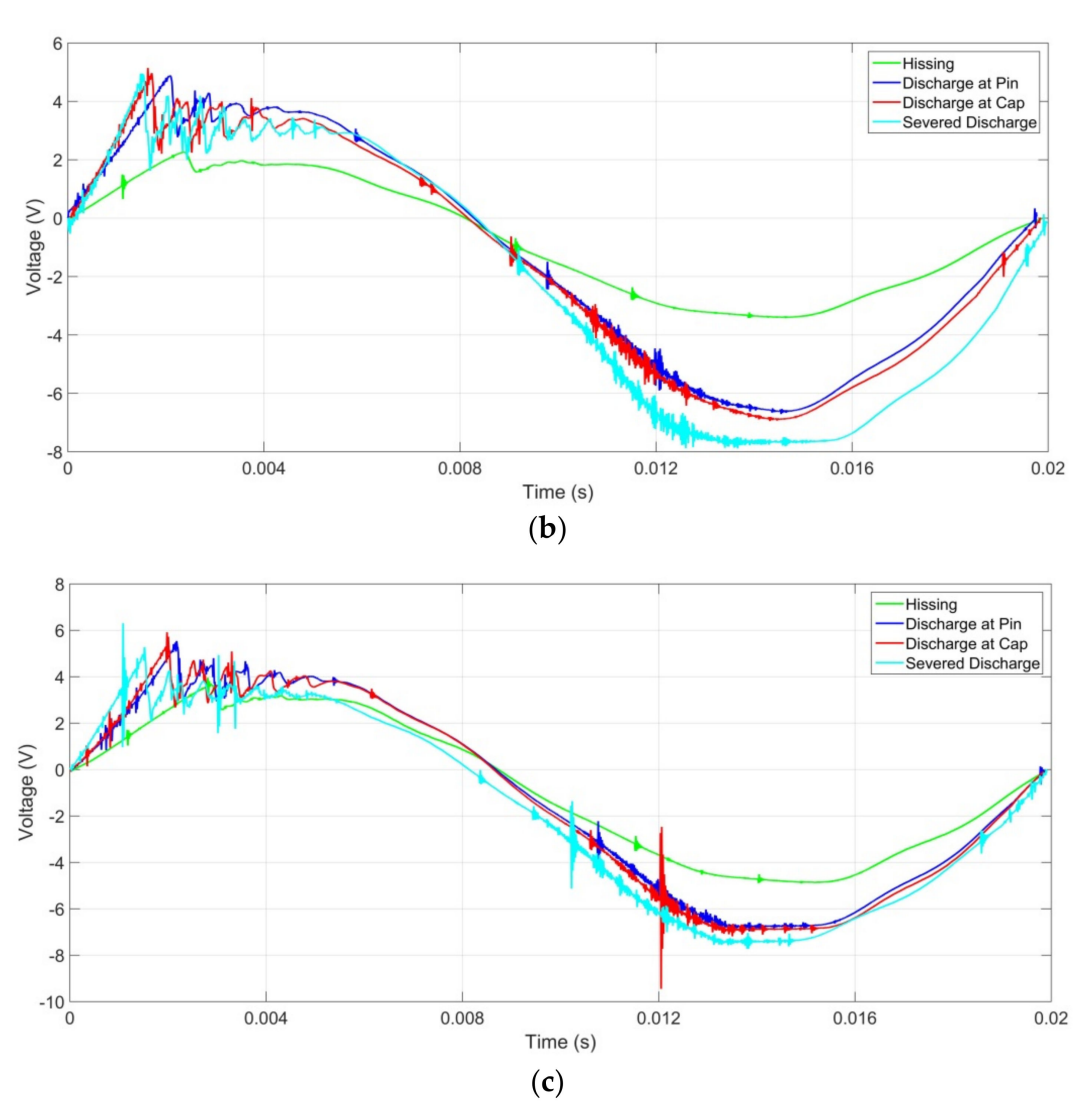
UV signal for insulator samples at medium contamination level with insulator ages of (**a**) <10 years, (**b**) 10 to 20 years, and (**c**) >20 years.

**Figure 8:**
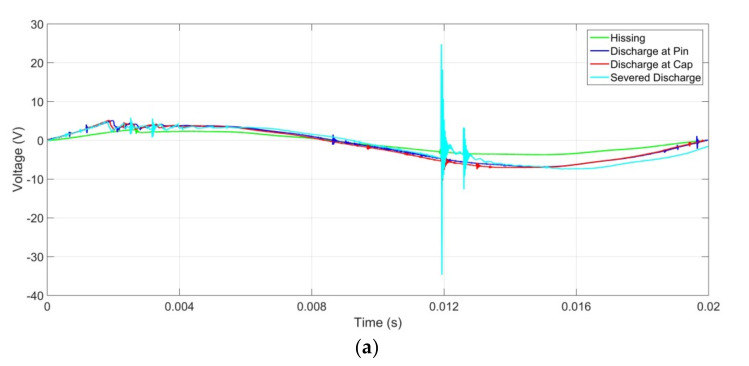

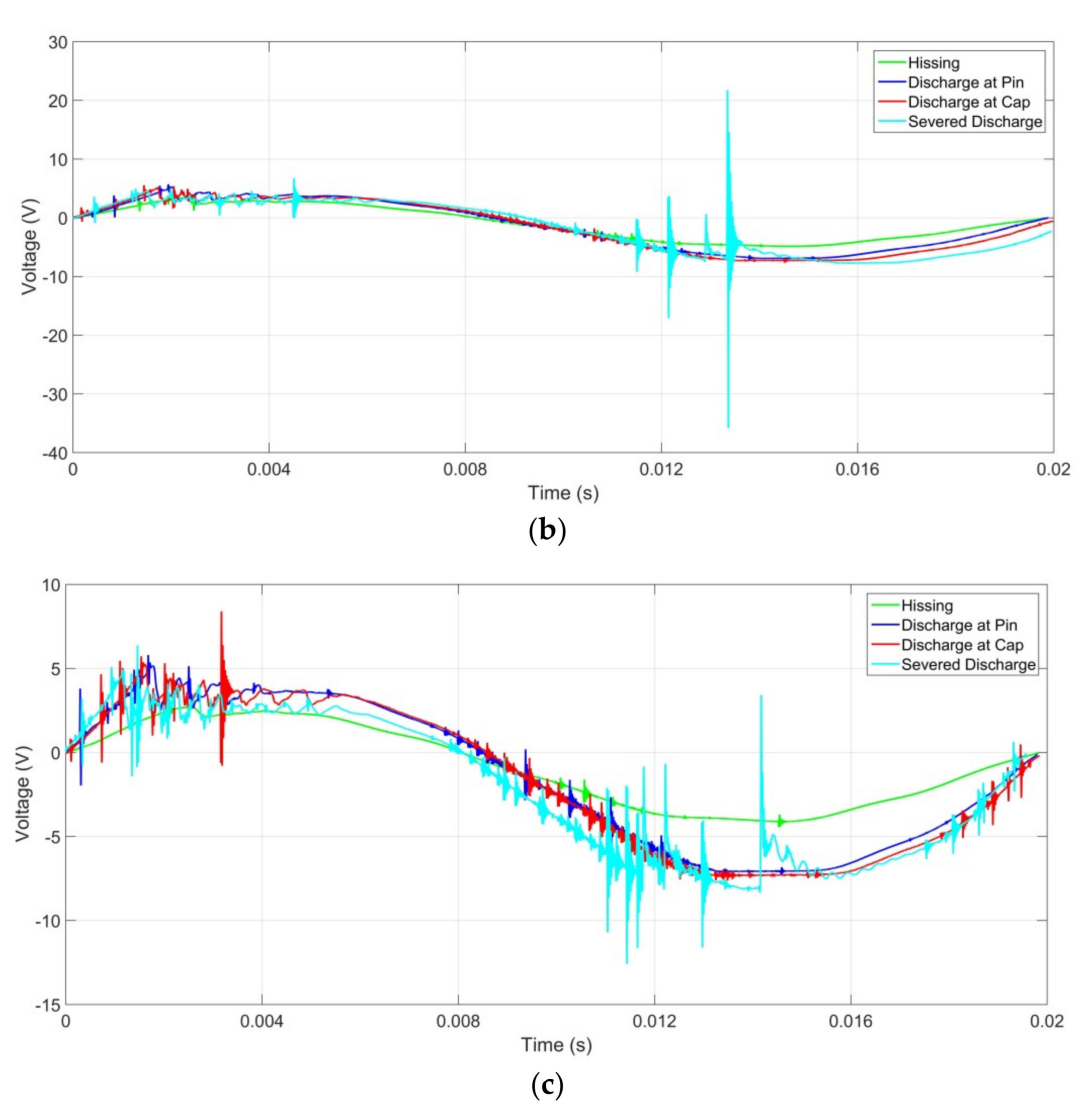
UV signal for insulator samples at heavy contamination level with insulator ages of (**a**) <10 years, (**b**) 10 to 20 years, and (**c**) >20 years.

The UV signals produced during discharge activities of the aged insulator samples and detected using the UV sensor under light, medium, and heavy contamination are presented in Figure 6, Figure 7 and Figure 8. These signals undergo signals processing to produce the harmonic component presented in this paper. The fundamental and harmonic components of these waveforms/signals were analysed using MATLAB, and the results were presented and discussed in the subsequent subsections.

### 4.1. DC Component Analysis

**Figure 9:**
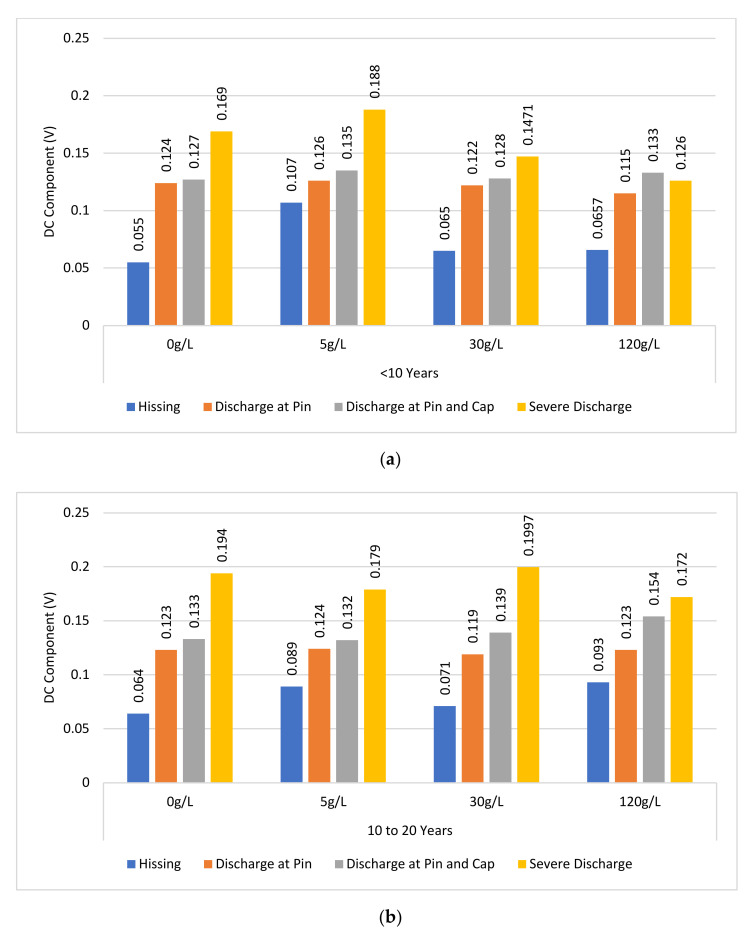

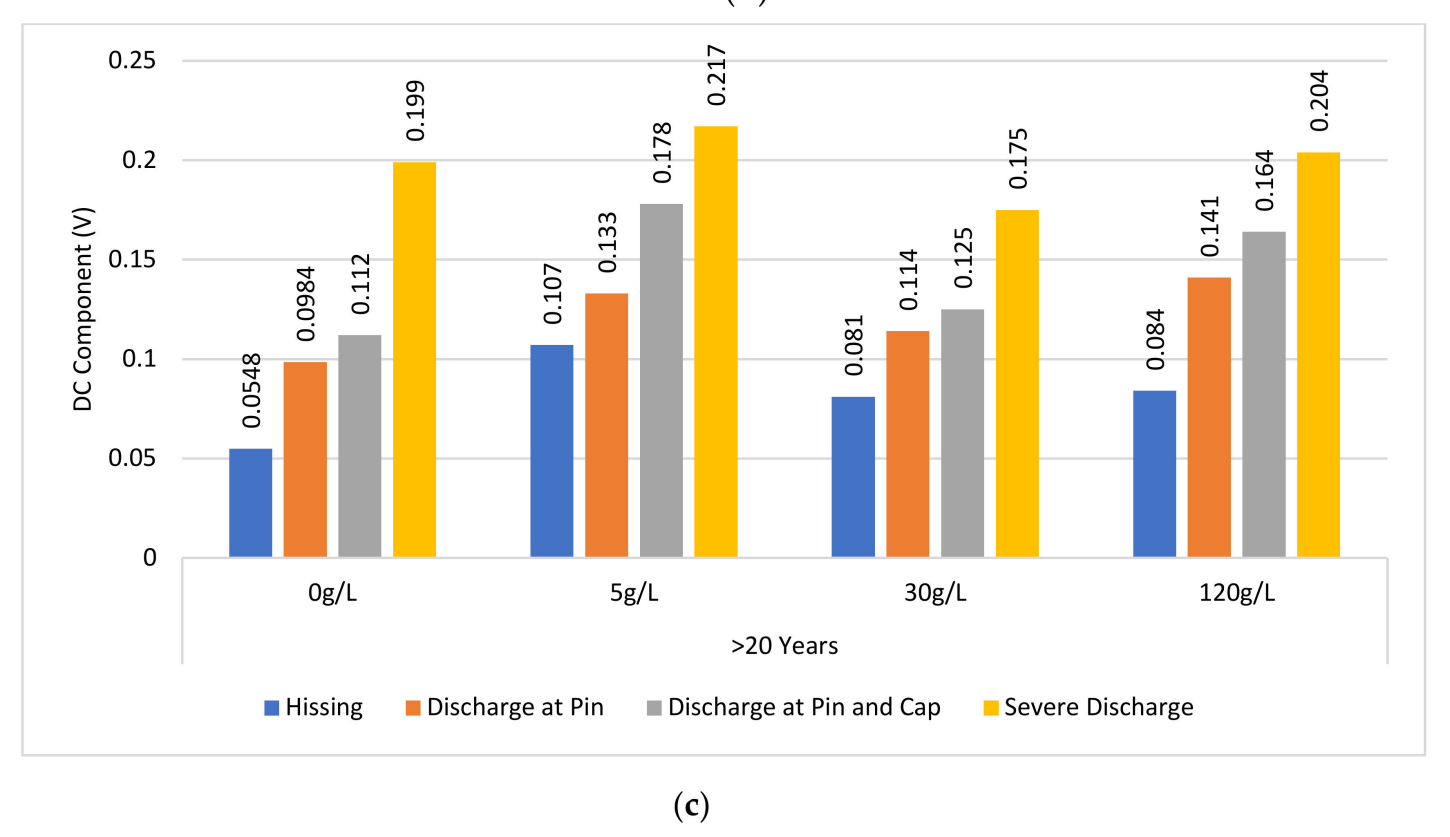
DC Component pattern for each level of contamination: (**a**) <10 years; (**b**) 10 to 20 years; (**c**) >20 years.

Figure 9 presents the DC components levels of the discharge intensity levels for each insulator age group under varying contamination levels. All measurements were repeated three times in order to ensure repeatability and low uncertainty in measurement. The results presented are the averages from the measurement produced.

#### 4.1.1. DC Component Based on Insulator Ages and Contamination

It can be observed from Figure 9 that there is generally an increasing trend in the DC components at each contamination level (none to heavy contamination) for almost all insulator ages (<10 to >20 years). As the electrical stress applied to the insulator increases, which increases the discharge intensity level, the DC component values increase.

There is a positive correlation between the DC components of the UV signal in the absence (0 g/L) or the presence (5–120 g/L) of contamination. Referring to Figure 9a (new insulators), in the absence of contamination (0 g/L), the DC components of the UV signal increased as the discharge intensity increased. The DC component UV signal was 0.055 V during hissing and increased to 0.169 V during severe discharge. This trend was also observed with the presence of a contamination level (5–120 g/L). Under light contamination level (5 g/L), the DC component level during hissing was 0.0809 V, and during severe discharge, it was 0.1673 V. Similarly, for aged insulator samples, Figure 9b,c the increasing trend of the DC components of the UV signals as the discharge intensity levels increased, were also observed for each contamination level.

However, based on the analysis of the insulator samples’ age, a strong positive correlation was not observed with regard to the relationship between the DC component levels of the UV signal and contamination levels at each discharge intensity level. Under clean surface condition (0 g/L), the DC component level for “hissing” discharge intensity level for new (<10 years), aged (10–20 years), and aged (>20 years) were 0.055 V, 0.064 V, and 0.055 V, respectively. Likewise, under medium contamination level, the DC component level for “Discharge at pin” discharge intensity level for new (<10 years), aged (10–20 years), and aged (>20 years) was 0.122 V, 0.119 V, and 0.114 V, respectively. This trend was observed for most of the other DC components of the UV signal under the different contamination levels compared between the insulator sample groups. This is expected owing to the fact that in this study, experiments were carried out under a dry contamination state. The conductivity of contaminants in the dry state (constant electric stress) is low and almost the same irrespective of the contamination level.

#### 4.1.2. Contaminated Insulator Samples

**Figure 10:**
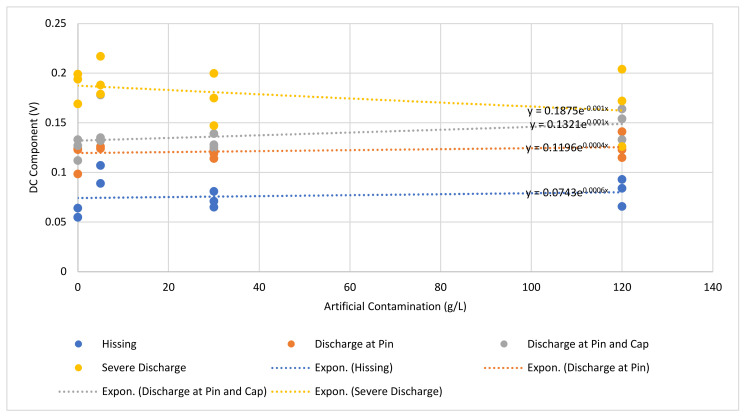
Average DC component for each level of discharge intensity at different contaminations levels.

Figure 10 shows a graph of the UV signal and DC signal component level for each discharge intensity level plotted for each artificial contamination level. The line of best fit for each discharge intensity level was also produced and presented in Table 5. From the equations, *y* is the DC component, and *x* is the contamination level.

Referring to Figure 10 and Table 5, it can be seen that there is a positive correlation between the DC components of the UV signal and the contamination level. For each particular discharge intensity level, the DC components of the UV signal increased with increasing contamination levels. Furthermore, there is also a strong positive correlation between the DC components of the UV signal and the discharge intensity. It can be seen from the graph, the DC component’s UV signal values for the “hissing” were the least, and the severe discharge was the highest.

#### 4.1.3. Aged Insulator Samples

**Figure 11:**
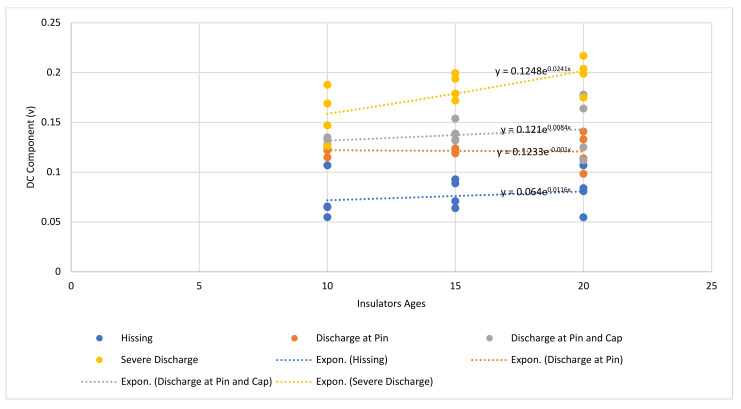
Average DC component for each level of discharge intensity at different insulator ages.

Figure 11 presents a graph of the DC component level of the UV signal for each discharge intensity level plotted for all three groups of aged insulator samples. The line of best fit for each discharge intensity level was also produced and presented in Table 6.

It can be seen from Figure 11 and Table 6 that there is a positive correlation between the DC components of the UV signal and the degradation level of the insulator samples. For each discharge intensity level, the DC components of the UV signal increased as the degradation level of the insulators increased. In addition, there is also a strong positive correlation between the DC components of the UV signal and the discharge intensity. It can be seen from the graph, the DC component’s UV signal values for the “hissing” were the least, and the severe discharge was the highest.

It can also be observed that the DC component UV signal values due to ageing (Table 6) are higher than those due to contamination levels (Table 5) under the same dry surface condition. This seems to suggest that under dry insulator surface conditions, the level of insulator degradation is of more concern than the level of contamination.

#### 4.1.4. Percentages Differences of the Insulator Samples’ Discharge Intensity Levels

**Figure 12:**
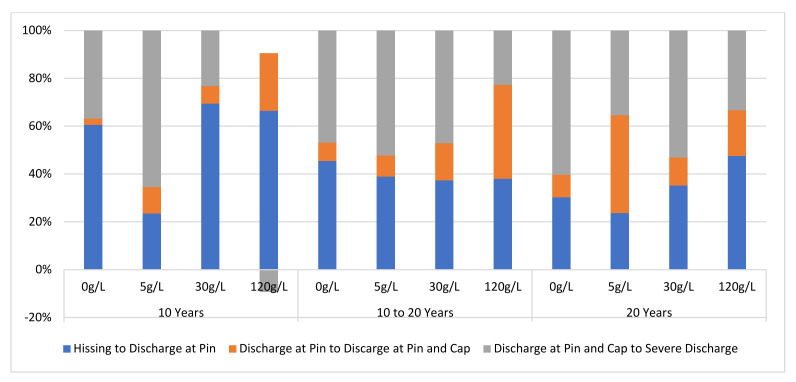
Average percentages are different between discharge intensity levels.

This section presents results of the percentage differences of the discharge intensity levels of the insulator samples under varying contamination levels and age. The DC signal components of the insulator samples’ DC signal components were used to compute the percentage differences. The percentage differences were grouped into three viz. percentage difference between hissing and discharge at the pin, the percentage difference between discharge at pin and discharge at cap, the percentage difference between discharge at cap and severe discharge. The results are presented in Figure 12.

From Figure 12. It can be seen that insulator ageing has a profound effect on the electrical stresses of the insulator samples’ surfaces under dry surface conditions compared to contamination levels. As the insulator samples age, higher intensity levels (discharges at cap and pin and intense discharges) dominate the insulator surfaces. This is indicated by the increase in the UV signals measured. As the electrical stresses of insulator samples groups of 10–20 years and >20 years increased, higher discharge intensity levels (discharges at cap, pin, and severe discharge intensity levels) dominated the insulator samples’ surfaces.

### 4.2. UV Signal Harmonic Component

This section presents the results and analyses of the harmonic components of the UV signal. The 2nd to 7th harmonic component of the UV signal was measured, and results were presented in the subsequent subsections.

#### 4.2.1. Contaminated Insulator Samples

**Figure 13:**
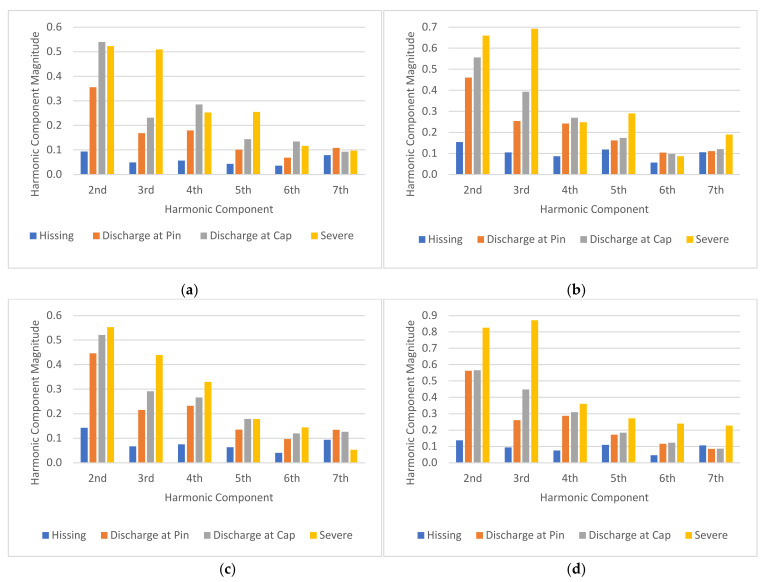
Harmonic component magnitude of each insulator contamination level: (**a**) 0 g/L, (**b**) 5 g/L, (**c**) 30 g/L, and (**d**) 120 g/L.

Figure 13 shows the plot of the 2nd to 7th harmonic component magnitudes of the insulator discharge intensity UV signal for the four contamination levels used in this study. The results of the harmonic components were obtained from the same insulator samples reported in the previous section. From Figure 13, it can be seen that under all the contamination levels (Figure 13a–d), there is a good correlation between the magnitudes of all harmonic components and the discharge intensities of the insulators except the 7th harmonic. The UV signal harmonic magnitudes increased with increasing discharge intensities. Furthermore, there is a good correlation between the magnitudes of the harmonic components and the contamination levels. Referring to Figure 13a, it can be seen that under clean (none) contamination, the 2nd harmonic magnitude under the “hissing” state was close to less than 0.1. As the contamination level increased, the 2nd harmonic magnitude also increased. Under Heavy contamination level, the 2nd harmonic magnitude in the “hissing” condition was close to 0.15. This trend is also observed for “discharge at pin,” “discharge at cap and pin,” and “severe discharge” states under all contamination levels in almost all harmonic components considered in this study except the 7th harmonic. The magnitude of the 2nd to 6th harmonic components had a strong correlation with the discharge intensities under various contamination levels.

#### 4.2.2. Aged Insulator Samples

**Figure 14:**
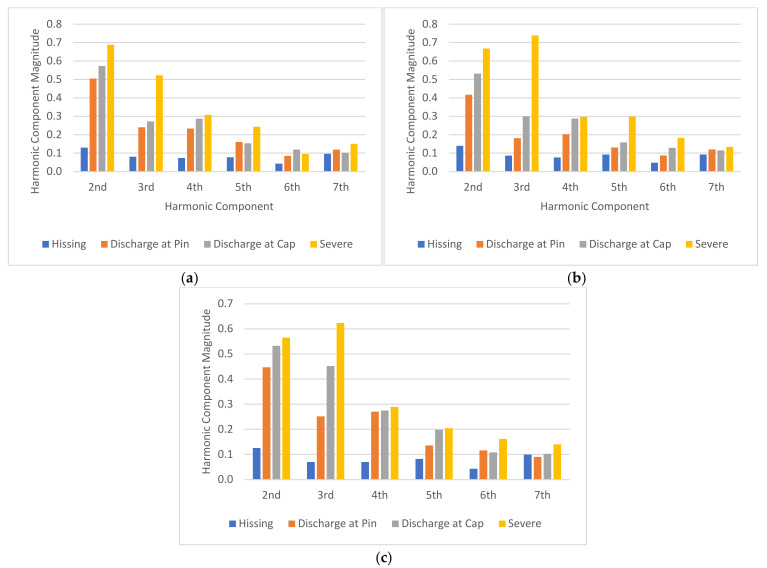
Harmonic component magnitude based on insulator ages: (**a**) <10 years, (**b**) 10 to 20 years, and (**c**) >20 years.

Figure 14 shows the plot of the 2nd to 7th harmonic component magnitudes of the insulator discharge intensity UV signal for the three classes of insulators having a different degree of aging (degradation) used in this study. From Figure 14, it can be seen that there is a good correlation between the magnitudes of all harmonic components and the discharge intensities among each of the classes of the insulators ages (Figure 14a–c) except the 7th harmonic. The UV signal harmonic magnitudes of the aged insulators generally increased with increasing discharge intensities. Referring to the insulators which have been in service for more than 20 years (Figure 14c), it can be seen that the 2nd harmonic component magnitude under the “hissing” state was 0.12. As the discharge intensities increased, the magnitude of the 2nd harmonic increased as well. The 2nd harmonic magnitude was 0.57 during the “severe discharge” state. This increasing trend was similar for all the harmonic components except the 7th harmonic.

#### 4.2.3. Average Harmonic Component of Insulator Samples

**Figure 15:**
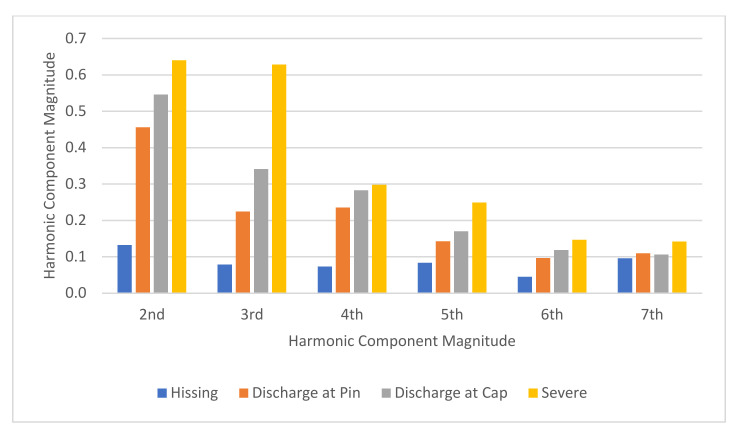
The harmonic component magnitude for each discharge intensity level.

Figure 15 presents the averaged harmonic component magnitude of the insulator samples. This result was obtained by combining and averaging the UV signal 2nd to 7th harmonic components of both contamination level and age at each discharge intensity level. It can be seen that there is a strong correlation between the harmonic component magnitude and discharge intensity levels at each harmonic component except the 7th harmonic. As the discharge intensity level increased, the harmonic component magnitude increased as well.

## 5. Conclusions

The DC and harmonic components of UV pulse signals detected using a UV pulse sensor due to the discharges on dry insulator surfaces under varying contamination levels, and insulator ages have been investigated in the work. The finding proved that UV pulse detected signal also gives a strong correlation between the discharge intensities and the degree of contamination and ageing as found by other methods as published before. The DC components of the UV pulse signals detected during discharge activities increased as the degree of contamination increased. Likewise, the discharge activities also increased as the insulator samples’ age increased. Furthermore, it is observed that under dry insulator surface conditions, insulator ageing has a more profound effect on insulators compared to contamination. Similar correlations were observed with the harmonic components of the UV pulse signals. As the discharge intensity level increased, the magnitude of the harmonic component also increased. This strong correlation between DC/harmonic components of the UV pulse signals and insulator contamination level/ageing can be a promising non-contact method of monitoring insulator surface discharges using UV pulse sensor as a predictive maintenance method/condition-based monitoring of transmission line insulators against flashover in the field.

## Figures and Tables

**Table 1:** Characterisation of the generated discharge intensity levels categories [12].

Discharge Intensity Level	Characteristics
Hissing	Hissing without any visible discharge
Discharge at pin of the insulators	Hissing sound plus spot discharges at the pin of the insulators
Discharge at cap and pin of the insulators	Louder hissing noise, discharges at both the pin and cap of the insulator samples
Severed discharge	Very loud hissing noise, intense sparking discharge on the pin and cap of the insulator (just prior to flashover)

**Table 2:** UVTRON R2868 sensor specification.

Parameter		Description/Value	Unit
General	Spectral Response	185 to 260	nm
	Window Material	UV glass	-
	Weight	Approximate 1.5	
Maximum Rating	Supply Voltage	400	V
	Peak Current	30	mA
	Average Discharge Current	1	mA
	Operating Temperature	−20 to +60	°C
Characteristics (at 25 °C)	Discharge Starting Voltage (with UV radiation) (DC)	280	V
	Recommended Operation Voltage (DC)	325 ± 25	V
	Background	10	min^−1^
	Sensitivity	5000	min^−1^

**Table 3:** Description of insulator samples.

Insulator Sample	Descriptions
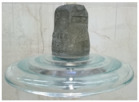	a) Insulators Age: Less than 10 Years b) Condition: Good
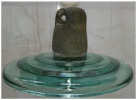	a) Insulators Age: 10 to 20 Years b) Condition: Mild corrosion at cap
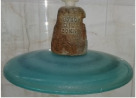	a) Insulators Age: More than 20 Years b) Condition: Discoloration of glass dielectric, severely corroded cap and pin

**Table 4:** Artificial contamination.

Salt (g/L)	ESDD (mg/cm^2^) [15]	Contamination Level [18]
0	N/A	None
5	0.06	Light
30	0.21	Medium
120	0.47	Heavy

**Table 5:** Exponential equation for each discharge intensity level with respect to insulator samples’ contamination level.

Discharge Intensity Level	Exponential Equation
Hissing	y = 0.0743e^0.0006x^
Discharge at Pin	y = 0.1196e^0.0004x^
Discharge at Pin and Cap	y = 0.1321e^0.001x^
Severe Discharge	y = 0.1875e^−0.001x^

**Table 6:** Exponential equation for each discharge intensity level with respect to insulator samples’ age.

Discharge Intensity Level	Exponential Equation
Hissing	y = 0.064e^0.0116x^
Discharge at Pin	y = 0.1233e^−0.001x^
Discharge at Pin and Cap	y = 0.121e^0.0084x^
Severe Discharge	y = 0.1248e^0.0241x^

## Data Availability

Not applicable.
